# Utilization of machine learning to predict antibiotic resistant event outcomes in acute myeloid leukemia patients undergoing induction chemotherapy

**DOI:** 10.3389/fcimb.2025.1629422

**Published:** 2025-08-21

**Authors:** Stephanie McMahon, Samantha Franklin, Jessica Galloway-Peña

**Affiliations:** Laboratory of Jessica Galloway-Peña, Texas A&M University, Department of Veterinary Pathobiology, Interdisciplinary Graduate Program in Genetics and Genomics, College Station, TX, United States

**Keywords:** antibiotic resistance, acute myeloid leukemia, random-forest, microbiome, resistome

## Abstract

**Introduction:**

Acute myeloid leukemia (AML) patients are highly susceptible to infection. Moreover, prophylactic and empirical antibiotic treatment during chemotherapy disrupts the gut microbiome, raising the risk for antibiotic-resistant (AR) opportunistic pathogens. There is limited data on risk factors for AR infections or colonization events in treated cancer patients, and no predictive models exist. This study aims to combine metagenomic and antibiotic administration data to develop a model predicting AR event outcomes.

**Methods:**

Baseline stool microbiome, antibiotic administration, resistome, and clinical metadata from 95 patients were utilized to build a Random Forest model to predict AR infection and colonization events by serious AR threats. Additionally, sparse canonical correlation analysis assessed correlations between microbiome and resistome data, while Spearman correlation networks identified direct associations with AR event outcomes and secondary variables.

**Results:**

AR-events were identified in 14 of the 95 included patients, with 8 developing AR infections and 9 identified as AR colonized. A Random Forest model predicted AR event outcomes (AUC = 0.73), identifying bacterial taxa and antibiotic resistance gene (ARG) classes as key variables of importance. *Methanobrevibacter smithii, Clostridium leptum*, and *Bacteroides dorei* were identified as key taxa associated with reduced risk of AR events, suggesting the potential roles of commensals in maintaining gut microbial resilience during chemotherapy. ARG classes, particularly those conferring resistance to lincosamides, macrolides, and streptogramins, were negatively associated with AR events.

**Conclusion:**

These results underscore the value of integrating microbiome and resistome features to reveal potential protective mechanisms and improve risk prediction for AR outcomes in vulnerable patients.

## 1 Introduction

Infections caused by multidrug-resistant organisms (MDROs) represent a major public health challenge, emphasizing the urgent need to understand the underlying factors contributing to antimicrobial resistance (Caniça et al., 2015; Castro-Sánchez et al., 2016; Palmore and Henderson, 2013). This understanding is essential for reducing infection rates and preserving the effectiveness of existing antimicrobial therapies (Caniça et al., 2015; Castro-Sánchez et al., 2016; Palmore and Henderson, 2013). The growing prevalence of antibiotic-resistant bacteria jeopardizes the effectiveness of commonly used antibiotics, complicating the treatment of an increasing number of infections and necessitating immediate action (Antimicrobial resistance, 2023; Walsh et al., 2023). The Centers for Disease Control and Prevention (CDC) has identified several pathogens as “urgent,” “serious,” and “concerning” antimicrobial-resistant (AR) threats in their 2019 Antibiotic Resistance Threats report (CDC, 2019). Among these, five pathogens are categorized as urgent threats, including methicillin-resistant *Staphylococcus aureus* (MRSA), multidrug-resistant *Pseudomonas aeruginosa*, vancomycin-resistant *Enterococcus* (VRE), extended-spectrum beta-lactamase-producing *Enterobacteriaceae* (ESBL), and carbapenem-resistant *Enterobacteriaceae* (CRE) (CDC, 2019). If left unrestrained, AR pathogens are projected to become the leading cause of death by 2050 (O’Neill, 2016).

Increasing evidence has underscored the essential role of the microbiome in defending against colonization and infection by antibiotic-resistant pathogens (Buffie and Pamer, 2013; Lewis et al., 2015; Ubeda et al., 2013). In addition to the microbiome defending against AR pathogens through colonization resistance, it also plays a role in immunomodulatory functions that influence infections in distant body sites (Belkaid and Hand, 2014; Latorre et al., 2015; Lewis et al., 2015; Panwar et al., 2021; Pickard et al., 2017; Thaiss et al., 2016). The widespread use of broad-spectrum antibiotics exerts selective pressure on microbial populations, facilitating the emergence of resistant strains (Chinemerem Nwobodo et al., 2022). Specifically, antibiotic treatment can disrupt the gut microbiota, leading to reduced microbial diversity, depletion of beneficial species, and the promotion of antibiotic-resistant gene (ARG) proliferation (Becattini et al., 2016; Gibson et al., 2015; Korpela et al., 2016; Modi et al., 2014). Moreover, overuse of antibiotics is known to not only facilitate the growth of MDROs, but also promote the horizontal transfer of resistance genes among organisms within the microbiota (Korry et al., 2020). This transfer can lead to the emergence of new multidrug-resistant pathogens, potentially restricting treatment options for bacterial infections. As a result, studies that explore the protective and harmful roles of the microbiota, the resistome (the collection of antibiotic resistance genes within the microbiota), and the specific impacts of antibiotic treatments on microbial communities contributing to the rise of antibiotic resistance are vital for developing new strategies to combat antimicrobial resistance (Caniça et al., 2015).

Patients with hematological malignancies are especially vulnerable to a wide range of infections, particularly those caused by AR pathogens (Gedik et al., 2014). Life-threatening infections are common, particularly in individuals who are immunocompromised with chemotherapy-induced cytopenia (Rapoport et al., 2021). Febrile neutropenia is especially prevalent in patients with acute myeloid leukemia (AML), affecting roughly 80-90% of individuals (Hansen et al., 2019; Taur and Pamer, 2016). To manage these risks, fluoroquinolone prophylaxis is routinely used before and during chemotherapy, and broad-spectrum empirical therapy is administered upon the onset of neutropenic fever following established standards of care (Boccia et al., 2022; Chemotherapy-Induced Neutropenia and Febrile Neutropenia in the US: A Beast of Burden That Needs to Be Tamed? | The Oncologist | Oxford Academic, n.d.; X. Wang et al., 2024; Zimmer and Freifeld, 2019). However, the prolonged use of antibiotics in these patients has been shown to disrupt their gut microbiota, leading to the accumulation of antimicrobial resistance genes (ARGs) (Aitken et al., 2021; Doan et al., 2020; Galloway-Peña et al., 2016; J. Galloway-Peña et al., 2017; Galloway-Peña et al., 2020; Iwan et al., 2024; Jutkina et al., 2016; Nobel et al., 2015). Chemotherapy also exacerbates the risk of AR infection by damaging the gut mucosa, increasing intestinal permeability, and facilitating the translocation of potentially resistant pathogens from the gut into the circulation, thereby heightening the risk of difficult-to-treat systemic infections (Sougiannis et al., 2021; Touchefeu et al., 2014).

The gut resistome is of particular significance in hospitalized cancer patients, as it serves as a key reservoir of ARGs that should no longer be overlooked when examining AR infectious complications (Gibson et al., 2014; Shono et al., 2016). The gut microbiome contains a large pool of ARGs, and these genes can be transferred among bacterial species within the microbiota via horizontal gene transfer, which presents potentially serious consequences for infections originating from the microbiome as well as transmission in the hospital environment (Forslund et al., 2013; Gibson et al., 2015; Pehrsson et al., 2013). Bacteria may carry ARGs that confer resistance to a single antibiotic or mobile genetic elements (MGEs) that provide resistance to multiple antibiotics. As the selection pressure for these bacteria increases, so does the number of bacteria harboring ARGs or MGEs, amplifying the resistome and resulting in more difficult-to-treat infections (Nielsen et al., 2021). Common bacterial culprits in infections among AML patients include the same key AR-threat pathogens identified by the CDC (VRE, MRSA, ESBL-producing *Enterobacteriaceae*, CRE, and multidrug-resistant *P. aeruginosa*) (On et al., 2022; Rolston, 2014). Although it is well-documented that cancer patients are frequently colonized and infected by AR pathogens, there is still a paucity of comprehensive data regarding the risk factors associated with AR infections and toxicities during chemotherapy (McMahon et al., 2023; Nanayakkara et al., 2021). This gap in knowledge underscores the need for further research to better understand the complex dynamics of the microbiome and AR infections in this vulnerable patient population.

In this study, we developed a machine learning model integrating patient antibiotic administration records with baseline fecal microbiome and resistome data. This model allowed us to identify and rank predictors associated with AR events in AML patients undergoing remission induction chemotherapy (IC). Additionally, sparse canonical correlation analysis was used to determine correlations between baseline ARGs and microbial taxa. A final network analysis was performed to identify variable connectivity with development of an AR event, and the directionality in which those variables relate to the event.

## 2 Methods

### 2.1 Study design and participants

Longitudinal stool samples and clinical data were collected from two cohorts of adult AML patients undergoing IC at MD Anderson Cancer Center (MDACC) between September 2013 and February 2020, for a total of 154 patients. The first cohort, PA13-0339, comprised 98 AML patients enrolled between September 2013 and August 2015, providing 566 stool samples collected as previously described (Galloway-Peña et al., 2016, 2020; J. R. Galloway-Peña et al., 2017) The second cohort, PA15-0780, included 56 adult AML patients enrolled from January 2015 to February 2020, contributing 216 stool samples. For this cohort, the stool sample taken within approximately one week of the start of IC was considered the baseline sample. Longitudinal samples were collected twice a week for the first four weeks, weekly for weeks four to eight, every other week for weeks eight to twelve, and then every two weeks after, continuing until either 24 weeks or loss of follow-up. Patients with missing baseline stool samples were excluded from the study. Between both cohorts, the average and median time of collection for a baseline sample was one day prior to chemotherapy initiation. A histogram depicting the time from baseline stool sample collection to initiation of chemotherapy is shown in **Supplementary Figure 1**. Patients from these two cohorts were only included in the analyses and model if they had complete metadata and sequencing data, which included baseline stool metagenomics, baseline gut resistome data, complete antibiotic administration data for the entirety of the study period, and the clinical factors of gender, chemotherapy type, and chemotherapy intensity available. This left 95 total patients with all data available to be included in the study.

### 2.2 16S rRNA sequencing and analyses of the stool samples

Genomic DNA was extracted from longitudinal stool samples using the QIAamp Fast DNA Stool Mini Kit (Qiagen), with modifications to the standard protocol that included an additional bead-beating lysis step. Each stool sample was placed in a tube containing a 3.2-mm steel bead, approximately 150 mg of zirconium beads, and lysis buffer. The samples were then homogenized using a bead-beater at 3800 rpm for 8 minutes (BioSpec) to facilitate DNA isolation. Amplicon libraries targeting the 16S V4 region were generated, and Illumina MiSeq sequencing was conducted on the fecal microbial DNA using a 2 × 250 bp paired-end protocol (Galloway-Peña et al., 2016, 2020; J. R. Galloway-Peña et al., 2017). A no-template control was used during the PCR, and a no-sample control for the extraction. The resulting reads were merged, dereplicated, and length-filtered using VSEARCH. Denoising and chimera detection were performed with the UNOISE3 commands, and the unique sequences, also known as zero-radius Amplicon Sequence Variants (ASVs), were taxonomically classified using Mothur with the SILVA database (version 138) (Edgar, 2016; Schloss et al., 2009). Alpha and beta diversity metrics were calculated in QIIME 2. The 16S rRNA sequences from the PA13 cohort have been previously published and are available in the NCBI Sequence Read Archive under Bioproject IDs PRJNA352060 and PRJNA526551 (Galloway-Peña et al., 2016, 2020; J. R. Galloway-Peña et al., 2017). The 16S rRNA sequences from the PA15 cohort are deposited in the NCBI Sequence Read Archive under Bioproject number PRJNA1124986.

### 2.3 AR event identification

All longitudinal stool samples underwent 16S rRNA sequencing to identify those with ≥ 3% of their 16S rRNA reads mapping to genera associated with the CDC urgent threat antibiotic-resistant (AR) pathogens, including vancomycin-resistant Enterococci (VRE), carbapenem-resistant Enterobacteriaceae (CRE), extended-spectrum beta-lactamase-producing Enterobacteriaceae (ESBL), multidrug-resistant *Pseudomonas aeruginosa* (MDRP), and methicillin-resistant *Staphylococcus aureus* (MRSA). The 3% threshold was chosen as it was previously determined that patients with less than 3% of 16S reads mapping to an AR-threat-associated genera had very little likelihood of obtaining a positive culture of an AR pathogen on selective and differential media (McMahon et al., 2023). Stool samples with >3% of 16S rRNA reads mapping to *Enterobacteriaceae, Escherichia, Enterobacter, Acinetobacter, Klebsiella*, or *Pseudomonas* were then cultured on CRE or ESBL selective media (Hardy Diagnostics Cat. No G323 and G321). Samples with >3% reads of *Enterococcus* were plated on VRE media (Chromagar Cat. No VR952), while those with *Staphylococcus* reads were streaked on MRSA selective media (Hardy Diagnostics Cat. No G307BX). Any colonies that grew on the selective media were then sub-cultured onto BBL Trypticase Soy Agar with 5% Sheep Blood (BD Biosciences) for isolation of individual colonies, which were stored at -80°C.

Matrix-Assisted Laser Desorption/Ionization Time-Of-Flight Mass Spectrometry (MALDI-TOF) was used to identify bacterial species from the purified colonies (Bruker MALDI Biotyper). After species identification, antibiotic susceptibility testing (AST) was performed using the VITEK2 system (Biomerieux). The AST-GN69 and AST-XN06 cards were used for Gram-negative bacteria, while AST-GP75 was used for Gram-positive isolates. If any bacterial isolate from a stool sample was confirmed to be CRE, ESBL-producing Enterobacteriaceae, VRE, MDRP, or MRSA, the patient was classified as having confirmed AR colonization (ARC).

In addition, any infectious bacterial isolates identified by the clinical microbiology lab at MDACC during the study, while AML patients were enrolled, were stored at -80°C. The clinical microbiology lab was responsible for determining the bacterial species and antimicrobial susceptibilities of these isolates. If a patient was diagnosed by the lab with a microbially defined infection caused by CRE, ESBL-producing Enterobacteriaceae, VRE, MDRP, or MRSA, the patient was classified as having a confirmed AR infection (ARI). These two groups—patients with confirmed ARC and those with ARI—were combined and classified as having an “AR event” for this study.

### 2.4 Whole genome sequencing of AR-event bacterial isolates and shotgun metagenomic sequencing of baseline samples

Following species identification, DNA was extracted from the ARI or ARC bacterial isolates using the MasterPure Gram-positive DNA purification kit (Lucigen). The extracted DNA was then used to prepare sequencing libraries with the Illumina DNA Tagmentation Library Prep kit (Illumina, San Diego, CA, USA). To evaluate the quality of the prepared libraries, the Qubit bioanalyzer and the Qubit dsDNA HS Assay Kit (Invitrogen, Waltham, MA, USA) were utilized. Once assessed, the libraries were pooled and sent to the North Texas Genome Center for sequencing on the Illumina NovaSeq 6000 S4 flow cell, using a 150-base pair paired-end read protocol. Subsequent sequencing data analysis was conducted on the Grace computing cluster at Texas A&M University. The sequencing reads were down-sampled to six million read pairs per sample using Seqtk (v1.3) before being assembled with SPAdes (v3.14.1) under the “—isolate” parameter. Annotation of the assembled sequences was performed using the RAST toolkit-based pipeline on BV-BRC (https://www.patricbrc.org/). The bacterial isolate sequencing data have been archived in the NCBI Sequence Read Archive under Bioproject ID PRJNA1129516.

For baseline fecal DNA extraction for metagenomic purposes, a modified version of the Qiagen Blood and Tissue kit (Qiagen, Valencia, CA, USA) was employed. Each sample, consisting of 150 mg of frozen fecal material, was combined with 500 µL of sterile InhibitEx Buffer (Qiagen, Valencia, CA, USA), 150 mg of silicon beads (Lysing Matrix B, MP Biomedical), and an appropriate amount of 2.4 mm metal beads from a hard tissue grinding mix (VWR, Radnor, PA, USA). The samples were placed in sterile tubes and subjected to bead beating at 4.5 m/s for four minutes using the MP Biomedical FastPrep-24 Classic system. The resulting suspension was vortexed, heated to 95°C for seven minutes, and centrifuged at 15,000 rpm for three minutes. DNA was extracted from the supernatant by adding 20 µL of Proteinase K and Buffer AL, followed by vortexing. After incubation at 70°C for 30 minutes, 200 µL of 100% ethanol was introduced, and the mixture was inverted multiple times before being transferred to a DNeasy spin column for purification. The extracted DNA was stored at -20°C for further analysis.

For shotgun metagenomic sequencing of baseline stool samples, DNA libraries were prepared using the Illumina Nextera XT DNA Library Prep kit (Illumina, San Diego, CA, USA). The quality of the libraries was confirmed using the Qubit bioanalyzer (Invitrogen, Waltham, MA, USA). Once validated, the libraries were pooled and sequenced at the North Texas Genome Center using the Illumina NovaSeq 6000 S4 platform, following the same protocol as the bacterial isolates. Samples were down-sampled to 6 million read pairs to normalize sequencing depth across subjects and reduce potential batch-related artifacts. This depth is sufficient for reliable class-level detection of ARGs in gut metagenomes, particularly when using curated HMM-based classifiers (BugSeq). A no-sample control was utilized for the extraction. Spike-in standards were not used in this study. Batch effects were minimized using consistent extraction protocols, library preparation methods, and sequencing conditions. Assembly of shotgun metagenomic sequences was performed using MEGAHIT (v1.2.8) and metaSPAdes (v3.14.1). The relative abundance of bacterial taxa was determined using MetaPhlAn2 (v2.8.1). The assembled shotgun sequences were further binned and annotated through the RAST Binning Service (RBS) on PATRIC. The finalized shotgun metagenomic data have been deposited in the NCBI Sequence Read Archive under Bioproject IDs PRJNA1129514 and PRJNA1128111.

### 2.5 Resistome analyses

Taxonomic binning of assembled sequences from shotgun metagenomic data was performed using BugSeq (v4.0), following previously established methods (Chandrakumar et al., 2022; Fan et al., 2021; Gauthier et al., 2022). Contigs were aligned against a curated reference sequence database using minimap2, and alignment results were evaluated based on query coverage and average nucleotide identity (ANI) thresholds to determine taxonomic classification. Antimicrobial resistance (AMR) determinants were identified by screening taxonomic bins with BugSeqs AMR analysis (v4.0). Contigs were analyzed against a curated protein database containing over 6,500 sequences associated with AMR, using both threshold alignment and Hidden Markov Models to accurately classify gene alleles and families. Additionally, taxon-specific models for phenotypic AMR prediction, incorporating single-nucleotide variants, insertions, deletions, and other genetic markers of resistance, were applied to over 50 bacterial species. The resulting taxonomic binning data and AMR predictions were compiled into comprehensive reports for further analysis. Antibiotic resistance genes (ARGs) in this study were examined at the class level.

### 2.6 Machine learning/random forest model

A machine learning model was developed by incorporating baseline microbiome species abundances via shotgun metagenomic sequencing, antibiotic resistance gene (ARG) data, antibiotic administration data, for time from baseline sample to chemotherapy start date, and binary clinical variables such as sex, chemotherapy type, and chemotherapy intensity to predict AR-threat outcomes. The model employed a Random Forest algorithm, which leverages multiple decision trees to reach an outcome. This approach, an extension of the bagging method, incorporates both bagging and feature randomness to create a “forest” of decision trees. Several patients were excluded from the study. Reasons for exclusion included: lack of baseline stool sample, lack of antibiotic administration data, lack of complete clinical metadata, and lack of resistome data. This left 95 patients with complete metadata to be included in the study (**Table 1**). Of these, 14 patients experienced an AR event during induction chemotherapy, defined either ARC or ARI events. Antibiotic administration variables were only included in the model if they were received by at least 10% of patients (9 or more patients). Similarly, ARG classes from resistome data were only included if they were present in at least 10% of patients. Baseline microbiome data was filtered to an abundance of 0.001 and a prevalence of 10%.

**Table 1:** Characteristics of the patients included for AR event analysis.

Patient characteristics	AR-event			
	Yes	No	P-value	X^2^
Patient count, N (%)	14 (14.7)	81 (85.3)		
Infection	8			
Colonization	9			
Cohort, N (%)				
PA13-0339	6 (6.3)	56 (58.9)		
PA15**-**0780	8 (8.4)	25 (26.3)		
Sex, N (%)			0.592	0.288
Female	8 (57.1)	40 (49.4)		
Male	6 (42.9)	41 (50.6)		
Chemotherapy intensity, N (%)			0.66	0.193
High	9 (64.3)	47 (58.0)		
Low	5 (35.7)	34 (42.0)		
Chemotherapy type, N (%)			0.83	0.882
Fludarabine^2^	4 (28.6)	18 (22.2)	0.732	–
Non-Fludarabine (High)^2^	5 (35.8)	29 (35.8)	>.99	–
Hypomethylators^2^	4 (28.6)	21 (25.9)	>.99	–
Other (Low)^2^	1 (7.0)	13 (16.1)	0.685	–
Antibiotic administration^1^			0.843	8.815
Levofloxacin	8 (57.1)	63 (77.8)	0.101	2.692
Metronidazole^2^	3 (21.4)	10 (12.3)	0.4	–
Linezolid^2^	13 (92.9)	57 (70.4)	0.104	–
Meropenem	7 (50)	34 (42)	0.576	0.313
Cefepime	9 (64.3)	49 (60.5)	0.788	0.072
Amikacin	5 (35.7)	11 (13.6)	0.041	4.176
Trimethoprim-Sulfamethoxazole^2^	0 (0)	9 (11.1)	0.3475	–
Daptomycin	5 (35.7)	22 (27.2)	0.512	0.429
Piperacillin-Tazobactam	6 (42.9)	22 (27.2)	0.234	1.415
Cefpodoxime	5 (35.7)	23 (28.4)	0.579	0.308
Ciprofloxacin^2^	2 (14.3)	16 (19.8)	>.99	–
Tigecycline^2^	4 (28.6)	13 (16.0)	0.269	–
Minocycline^2^	3 (21.4)	9 (11.1)	0.377	–
Ertapenem^2^	2 (14.3)	16 (19.8)	>.99	–

^1^Antibiotic Administration is defined as any administration of the antibiotic.

^2^Fisher’s Exact test was used.

To minimize dataset variability, overfitting, bias and to enhance robustness, we ran four different models. For each model, analysis was performed across 100 independent 80–20 stratified training-testing splits. Model 1 contained 89 variables and featured ARG class by presence/absence, presence/absence of antibiotic administrations received ≥72 hours, and baseline species abundance from shotgun metagenomic sequencing. Model 2 also contained 89 variables but featured unique ARG counts by class, presence/absence of antibiotic administrations received ≥72 hours, and baseline species abundance. Model 3 contained 92 variables with ARG class by presence/absence, presence/absence of any administration (regardless of duration), and baseline species abundance. Finally, Model 4 also contained 92 variables, with unique ARG counts by class, presence/absence of any antibiotic administration, and baseline species abundance. Antibiotic administration was evaluated at a threshold of ≥72 hours of administration or as the presence/absence of any administration, regardless of duration, as both are biologically relevant for infectious implications in this patient cohort. AML patients receiving IC may receive empirical broad-spectrum antibiotic treatment, which might be de-escalated upon return of negative cultures (~48hrs) or switched to a separate antibiotic with the correct spectrum of activity if the pathogen is found resistant to the prescribed antibiotic. Thus, presence/absence would account for any antibiotic given empirically or otherwise, whereas a model that considers antibiotic administration ≥72 hours is relevant for antibiotics that were maintained/continued for an infectious implication (AR or not) after return of culture or continuation of symptomology.

The resulting outcomes of each iteration were then aggregated, either as ROC curves or box plots for each model. Variable importance scores for each of the 100 iterations were calculated and averaged. To better amend the model’s performance, the top 20, 15, and 10 most influential variables were selected for inclusion for each of the four models. The model with the 15 most influential variables demonstrated the best performance in comparison to 10 or 20 in all four models. Each model using the top 15 most influential variables was then executed 100 times, and the aggregated results were used to produce a final AUC-ROC curve. The variable importance scores were calculated as the raw sum of decreases in Gini impurity across all trees in the Random Forest and were not normalized. The Youden’s index was calculated as sensitivity plus specificity minus one to summarize the overall accuracy of the diagnostic test. A final model (Model 2) was chosen based on the 1) highest AUC, 2) best balance of sensitivity and specificity, and 3) a balanced Youden’s index. The final model was optimized by increasing the value or mtry to increase the number of variables randomly sampled in each try and increasing the node size to increase the depth of each tree. We then used Shapely Additive exPlanations (SHAP) to interpret the optimized machine learning model.

### 2.7 Sparse canonical correlation analysis

Sparse canonical correlation analysis (sCCA) was utilized to detect ARG class-microbial species associations by identifying highly correlated linear combinations of variables while ensuring sparsity through variable-specific weight constraints. A centered log-ratio (CLR) transformation was applied to the microbial abundance data, with zero values substituted by a pseudocount calculated as min(relative abundance)/2. Sparse Canonical Correlation Analysis (sCCA) was performed using the ‘PMA’ package (v. 1.2-2) in R, correlating the CLR-transformed gut microbiome taxonomic composition with the ARG classes. Hyperparameters were optimized using the CCA.permute function (nperms = 100, niter = 5) prior to model fitting. To enforce sparsity, a Lasso penalty was applied, with the “typex” and “typez” parameters set to “standard” for the corresponding canonical vector (Rashidi et al., 2022).

### 2.8 Network development and selection of variables

To investigate the interactions between classes of ARGs by count of unique genes within each class, antibiotic administration by presence/absence, microbial taxa abundance at the species level, and AR events, a network was constructed based on a correlation matrix derived from the sample data. The matrix was created using Spearman’s rank correlation test, with positive values indicating a positive correlation to the AR event and negative values indicating a negative correlation (Spearman, 1904). The resulting correlation matrix was imported into Gephi, where the network was visualized using its built-in functions (Bastian et al., 2009). Following the initial analysis, edges with a weight below 0.2 were removed, resulting in the identification of 11 variables associated with AR events. The nodes and edges were then uploaded to generate the network.

## 3 Results

### 3.1 Patient characteristics

During their inpatient treatment, 14 patients experienced an AR event. Of these, 8 patients were identified with antibiotic-resistant infections, with one patient exhibiting two distinct infections. Among the infections, 37.5% were attributed to carbapenem-resistant *Pseudomonas aeruginosa*, 12.5% to MRSA, and 62.5% to ESBL *Escherichia coli*. Furthermore, 9 patients were identified as being GI-colonized, with 22.2% of those colonized with VRE, 22.2% with carbapenem-resistant and ESBL-producing *E. cloacae*, 22.2% with ESBL-producing *Klebsiella pneumoniae*, 33.3% with MRSA, and 22.2% ESBL-producing *E. coli*. Of these 14 total AR event patients, 3 patients had overlapping calls, where they had both ARC and ARI (**Supplementary Table 1**). Analysis of clinical variables demonstrated that sex, chemotherapy type, and chemotherapy intensity did not show any significant differences between AR event groups (**Table 1**). The only variable that showed a statistical difference between the two groups was amikacin administration (p=0.041) (**Table 1**). These results ultimately show that there are no clinical confounding variables between the two AR event groups.

### 3.2 Development of model

**Figure 1:**
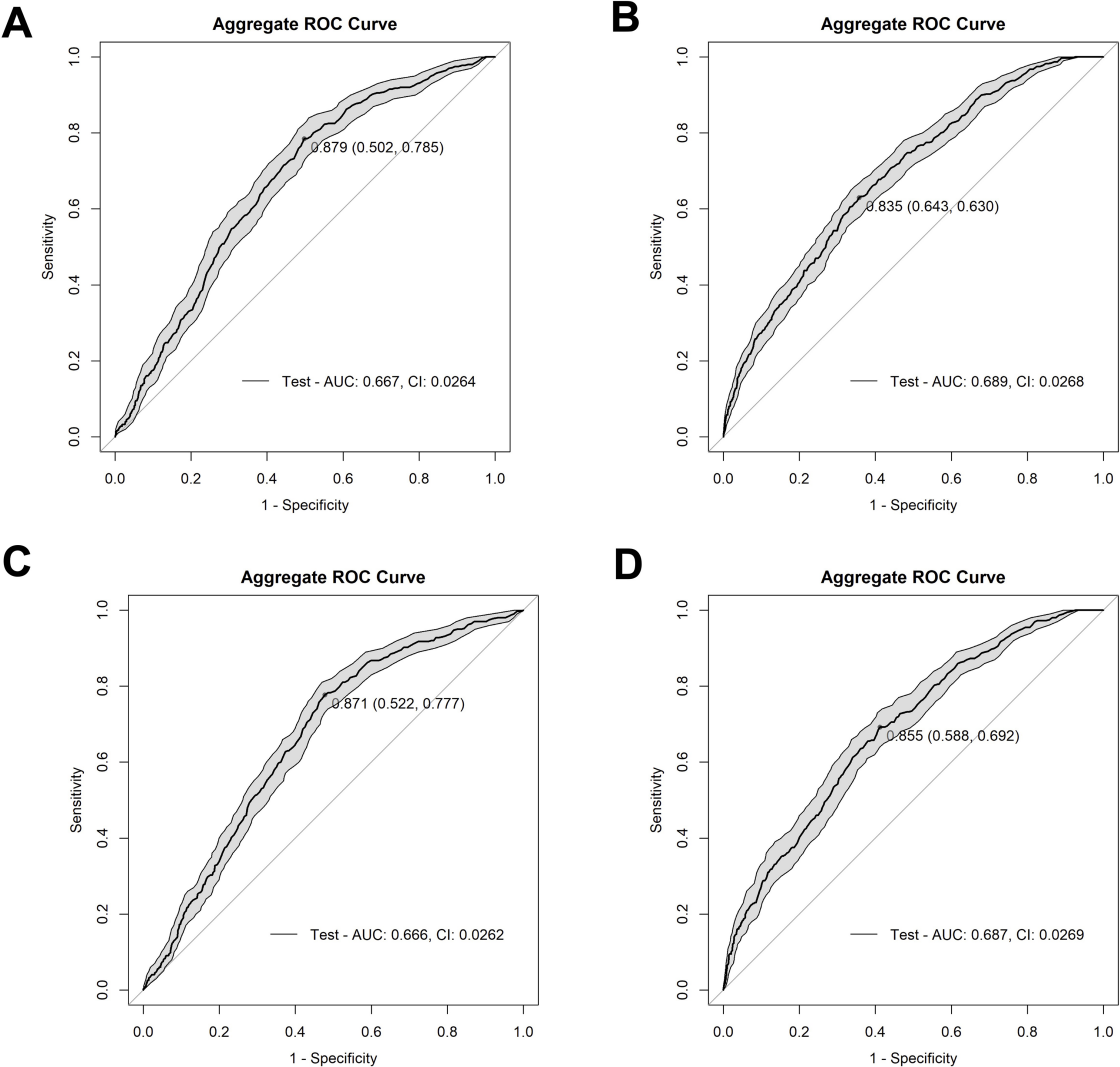
Receiver operating characteristic (ROC) curves depicting the performance of our four model methods. **(A)** depicts Model 1 (AUC=0.667) with ARG class by presence vs absence, presence/absence of antibiotics at ≥72 hours’ worth of administrations, and baseline species abundance, **(B)** depicts Model 2 (AUC=0.689) with unique ARG counts by class, presence/absence of antibiotics ≥72 hours of administrations, and baseline species abundance, **(C)** depicts Model 3 (AUC=0.666) with ARG class by presence vs absence, antibiotic administration presence/absence of any administration, and baseline species abundance, and **(D)** depicts Model 4 (AUC=0.687) with unique ARG counts by class, antibiotic presence/absence of any administration, and baseline species abundance. The shaded grey area on the graph shows confidence intervals for all models. The Youden’s Index, a metric that identifies the optimal threshold on a ROC curve, is indicated by the dot on the curve, followed by the specificity and sensitivity values, respectively.

To develop a model capable of predicting AR event outcomes, a Random Forest-based approach was employed to determine the optimal model configuration and identify the variables that most significantly contributed to the model’s performance. The patient data were randomly partitioned into a training set (80% of the samples) and a testing set (20% of the samples). Each model was trained over 100 independent iterations, each with 500 trees. Variable importance scores were calculated for each feature, and each model’s performance was assessed using the area under the curve (AUC) and Youden’s index, which calculates the maximum vertical distance between the curve of true positives and false positive rates on an ROC curve. After running each model with all variables (89–92 variables depending on the model), the top 15 variables for each model were selected based on their importance scores and the models subsequently re-run with only the 15 retained variables (80/20 split, 100 iterations, 500 trees each). The aggregated results were used to produce a final AUC-ROC curve for each model (**Figure 1**). Based on antibiotic administration data and antibiotic-resistant gene (ARG) data from BUGSEQ, we constructed four distinct model variations to assess which data configuration produced the most robust results. Model 1 incorporated ARG class by presence/absence, presence/absence of antibiotics administered ≥72 hours, and baseline taxa abundance by species (AUC=0.667, Youden’s=0.879) (**Figure 1A**). Model 2 included ARG data by count of unique genes by class, presence/absence of antibiotics administered for ≥72 hours, and baseline species abundances (AUC=0.689, Youden’s=0.835) (**Figure 1B**). Model 3 utilized ARG class presence/absence data, presence/absence of any antibiotic administered (regardless of duration), and baseline species abundance (AUC=0.666, Youden’s =0.871) (**Figure 1C**). Model 4 comprised ARG data by count of unique genes by class, presence/absence of any antibiotic administered, and baseline abundance data by species (AUC=0.687, Youden’s=0.855) (**Figure 1D**). Further clinical metrics and confusion matrices for each model can be found in **Supplementary Tables 2** and **3**, respectively.

**Figure 2:**
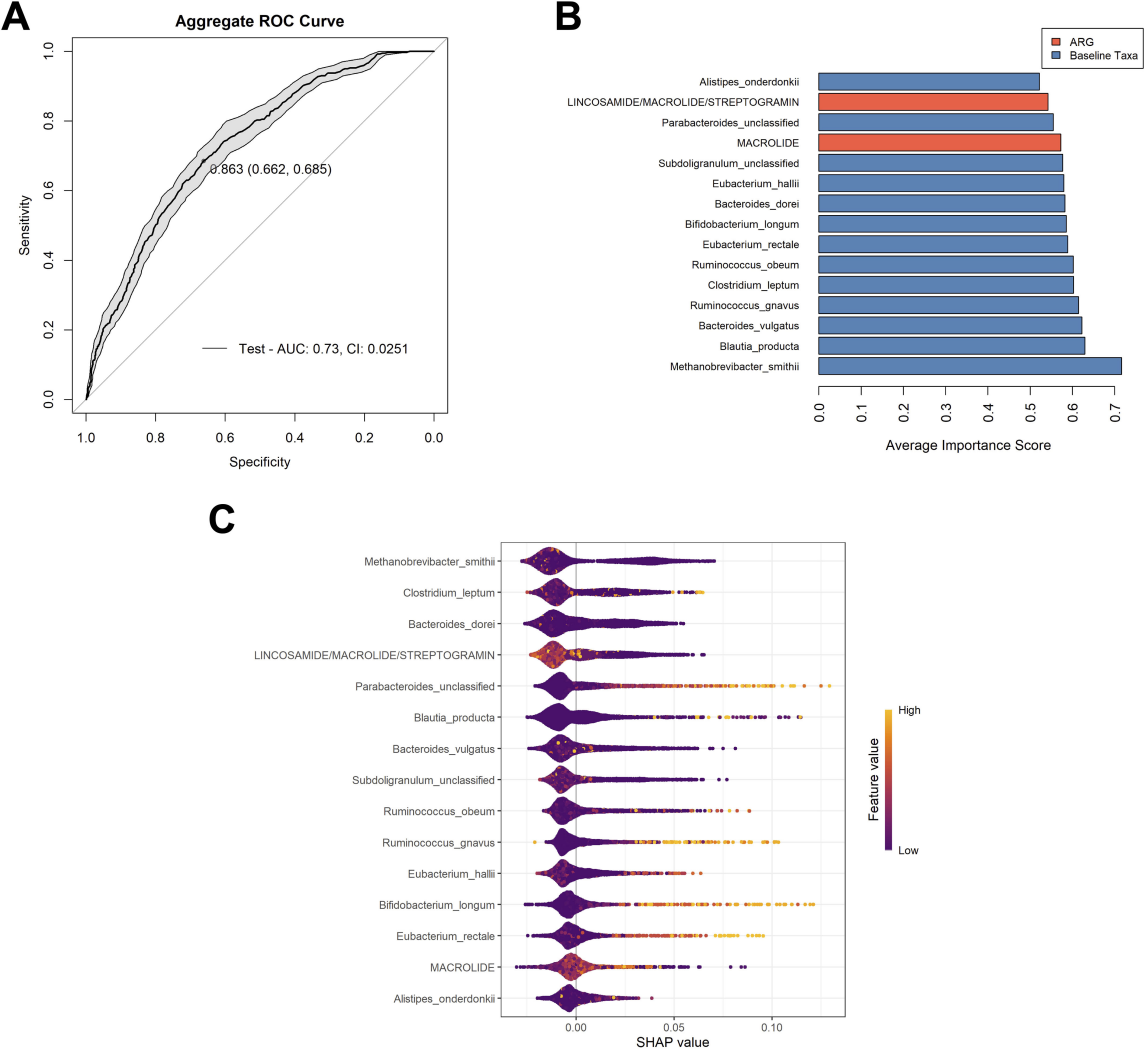
Analysis of the chosen model. **(A)** Aggregate ROC curve for Model 2 following optimization. The optimal cutoff point, determined by maximizing the Youden’s index is indicated on the curve, followed by the specificity and sensitivity, respectively. The shaded grey area on the graph shows the confidence interval for the model. **(B)** The top 15 variables used in the optimized model that had the highest contributions to model performance. Variable important scores are calculated by the mean decrease in node impurity (Gini importance). Variables are color coordinated with bacterial taxa in blue and ARG classes in orange. Most contributory variables are listed from most (bottom) to least contributory variables (top). **(C)** The SHAP (Shapely Additive exPlanations) beeswarm plot summarized the impact of each feature of the model’s predictions across all samples and aggregated runs. Each point represents a single sample’s SHAP value for a given feature, with the x-axis indicating the SHAP value (the effect of the feature on the model output for that sample). Features are ranked on the y-axis by their overall importance (mean absolute SHAP value). Color represents the original value of the feature for each sample (with lighter colors indicating higher values and darker colors indicating lower values). Points to the right indicate that the feature increases the predicted probability of the outcome for those samples, while points to the left indicate a decrease. The distribution and color gradient of the points reveal how high or low values of each feature influence the model’s predictions. The variables are listed in order of most contributory to the model’s prediction across all samples to least from top to bottom.

We determined that Model 2 was the best choice among the four models as it offers the strongest overall performance balance. It has the highest AUC (0.689), indicating the best discrimination between positive and negative cases. While its sensitivity (0.630) is slightly lower than the others, it achieves the highest specificity (0.643) among the models. Moreover, although models 1, 3, and 4 had higher sensitivity, their specificity was not much above 50%. Having determined that Model 2 was the most balanced model, the model was optimized, yielding an aggregate AUC value of 0.730 (CI: 0.0251), and a Youden’s index of 0.863 (**Figure 2A**). The aggregate optimized model had a mean AUC value of 0.742, a median AUC value of 0.745, and a range from 0.505 to 0.969 for the 100 iterations (**Supplementary Figure 2**).

Of the 15 variables utilized in this model, 13 were bacterial taxa, with *Methanobrevibacter smithii* (mean variable importance score (mVIS) of 0.717) and *Blautia producta* (mVIS of 0.630), being the most contributory to the model. Two classes of ARGs were also contributory to the model, those being genes predicted to encode resistance to macrolides (mVIS of 0.573) and genes classified as those predicted to encode resistance to lincosamides, macrolides, and streptogramin (mVIS of 0.542). The additional 11 variables consisted of bacterial taxa, with mean VIS scores ranging from 0.522 to 0.622 (**Figure 2B**).The SHAP method was used to explain the contribution or importance of each of the 15 features on the prediction of the model for AR events (**Figure 2C**). The predominant clustering of SHAP values below zero for each variable indicates that these features tend to have a negative impact on the prediction of an AR event, supporting a non-event classification. Specifically, higher values of ARG counts of genes belonging to macrolide and lincosamide/macrolides/streptogramin classes contributed negatively to the model. It also appeared that the higher feature values of *M. smithii*, *Clostridium leptum*, and *Bacteroides dorei* contributed negatively towards the model, which were the top three features with the greatest effect on prediction, according to the mean SHAP value. Conversely, it appeared that higher feature values of baseline abundances of *Bifidobacterium longum*, *Ruminococcus gnavus*, *Eubacterium rectale*, and *Parabacteriodes* (unclassified), contributed positively to the model.

### 3.3 Sparse canonical correlation analysis

To better understand the relationship between baseline species abundances and antibiotic resistance gene (ARGs) classes in patients at baseline, a sCCA analysis was conducted to explore the associations between these two groups. A heatmap was generated using Pearson correlation coefficients (**Supplementary Figure 3**). Notably, only 2 ARG classes and 10 taxa were found to be correlated between baseline gut microbiota species and classes of ARGs. Hierarchical clustering revealed four bacterial clusters, with two that were particularly noteworthy. The first cluster included *Streptococcus thermophilus* and *Bacteroides thetaiotaomicron*, which were negatively correlated with genes encoding resistance to glycopeptides and lincosamides/macrolide/streptogramins. In contrast, the second cluster revealed that *C. leptum* and *Ruminococcus torques* exhibited a positive correlation with genes conferring resistance to glycopeptides and lincosamide/macrolides/streptogramins.

### 3.4 Network development

**Figure 3:**
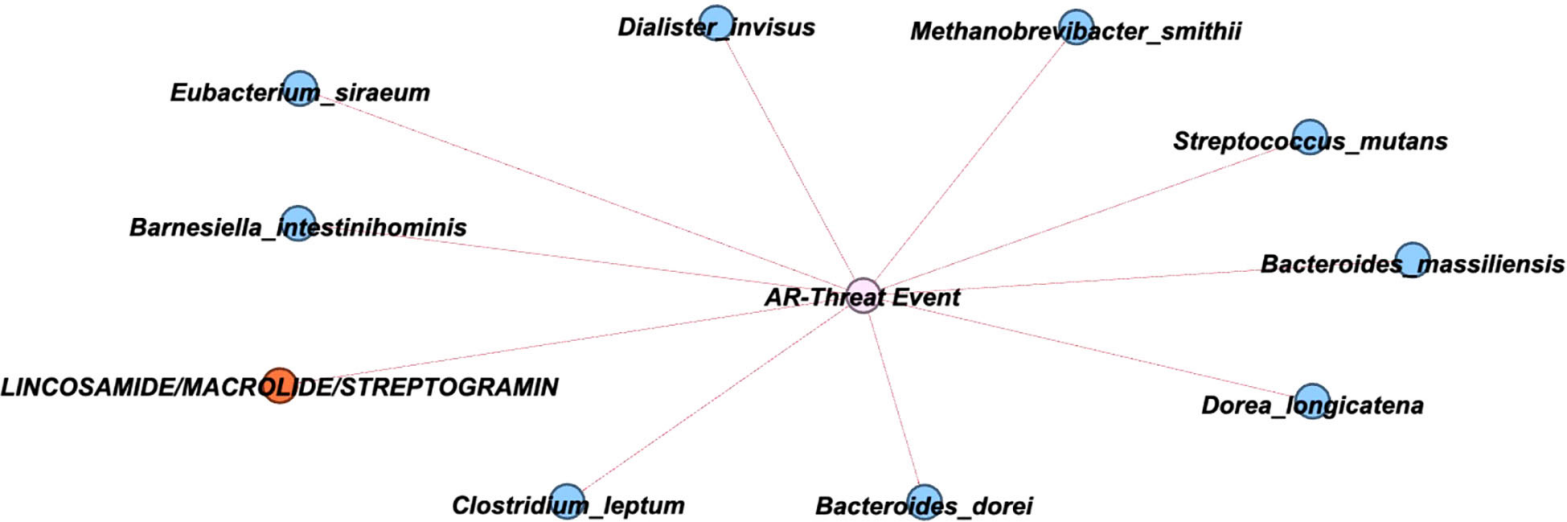
Network showing correlations with AR Event. A graphical network with primary nodes connected to the AR event. Within the network, the nodes are classified to denote bacterial species (blue), ARG classes (orange), with the event notated in the center (pink). The direction of connections is indicated to be negative by a red line.

Thus, to further comprehend how the variables relate to the development of an AR event, a network analysis was conducted to explore these correlations. After constructing a correlation matrix with a minimum correlation threshold of 0.2, 11 variables were identified as linked to the development of an AR event and visualized in a network (**Figure 3**; **Supplementary Table 4**). The network analysis suggests that a patient’s baseline taxa might play a critical role in protecting against an AR event, as all 11 variables were negatively correlated with AR event occurrence.

## 4 Discussion

In this study, we utilized a novel machine learning approach to predict antibiotic-resistant event outcomes in patients undergoing induction chemotherapy for acute myeloid leukemia. The developed Random Forest model integrated baseline fecal microbiome composition, antibiotic administration data, and resistome profiles, successfully identifying critical microbial taxa and antibiotic resistance gene classes associated with AR events. Our best performing model, which incorporated ARG class count data and antibiotics by presence or absence at ≥72 hours, yielded a promising predictive performance with an AUC of 0.730.

Across our integrated analyses, *M. smithii, C. leptum*, and *B. dorei* consistently emerged as key taxa associated with AR events during induction chemotherapy, underscoring their potential roles in shaping the gut ecosystem’s vulnerability to resistant pathogen colonization or infection. *M. smithii* was the top-ranked feature in the Random Forest model by both mVIS and SHAP values and determined to be negatively associated with AR events in the correlation network. This species commonly contributes to gut microbial homeostasis through hydrogen consumption and syntrophic interactions with fermentative bacteria, which can help maintain community stability during antibiotic-induced perturbation (Adrian et al., 2023; Ghavami et al., 2018; Malat et al., 2024). Prior studies have linked *M. smithii* to a more resilient gut environment and a reduced risk of inflammatory or infectious conditions, which are consistent with our findings of this species having a negative correlation with antibiotic-resistant event development (Chen et al., 2024; Cisek et al., 2024; Ghavami et al., 2018). Interestingly, the sCCA, a method designed to reveal coordinated variation between taxa and resistome features, depicted *M. smithii* showing weak positive correlations with several ARG classes, including those conferring resistance to lincosamides, macrolides, and streptogramins, which were also found to be negatively contributory to AR event prediction and correlated with AR events in the correlation network. This may suggest that *M. smithii* persists in communities where ARGs are present but well-regulated by stable microbial networks, preventing overgrowth of pathogenic species despite the elevated resistance gene abundance (Dongre et al., 2025; Nhu and Young, 2023).

*C. leptum* was also positively correlated with the ARG abundance of genes conferring resistance to glycopeptides and the lincosamides/macrolides/streptogramins via the sCCA, indicating this species tends to co-occur with these resistance elements in the gut ecosystem. Previous studies have similarly linked *C. leptum* to dysbiotic gut environments and long-term ARG retention following antibiotic exposure (Korpela et al., 2016; Nielsen et al., 2021). Yet despite these associations with elevated ARG content, *C. leptum* was shown to be negatively correlated with the development of an AR event in the correlation network, and negatively contributory to the Random Forest model via SHAP analyses. Rather than serving as a direct risk factor, its presence may reflect a type of microbiome that, while enriched for resistance genes, is still ecologically balanced enough to prevent extensive growth or translocation of pathogenic organisms (Grenda et al., 2022; Guo et al., 2020). This underscores the ecological nuance in the gut microbiome, where a species might contribute to resistance gene maintenance without necessarily increasing infection risk. Moreover, the presence of ARGs does not necessarily equate to virulence/pathogenesis of a pathogen, nor does it mean those genes are expressed. This also highlights the value of using multiple analytical approaches to uncover different layers of interaction between microbes, resistance elements, and clinical outcomes.

*B. dorei* was also identified as an important feature negatively contributory in the Random Forest model and demonstrated a negative correlation with AR event outcomes in the network analysis, suggesting that its presence may help guard against resistant pathogen colonization or infection. *B. dorei* is a prominent commensal species that contributes to gut health by reinforcing epithelial barrier integrity, producing immunomodulatory metabolites, and participating in the exclusion of pathogenic bacteria (Panwar et al., 2021; Pickard et al., 2017). These attributes become particularly relevant during chemotherapy, when the gut barrier is compromised, and antibiotic disruption leaves the microbiome vulnerable. The fact that *B. dorei* was shown to be negatively associated with the development of AR events suggests that it may help maintain gastrointestinal integrity or colonization resistance against pathogens under stress, possibly acting as part of a protective microbial buffer against the emergence or expansion of resistant organisms.

Among all the antibiotic resistance gene classes we examined, the group conferring resistance to lincosamides, macrolides, and streptogramins stood out for their consistent and unexpected association with reduced AR event risk. These genes showed up across each different analysis, 1) they were among the top predictors in the Random Forest model, suggesting a strong relationship with AR event risk; 2) they showed covariation with specific microbial taxa in the sCCA, indicating possible ecological linkage; and 3) they demonstrated a negative correlation with AR events in the network analysis. This last point is particularly unusual as resistance genes from this class are typically associated with broad-spectrum antibiotic use and worse clinical outcomes, especially in settings involving high-risk pathogens (Khodabandeh et al., 2019). One possibility as to why we are seeing these ARG classes negatively associated with AR events is that they might represent intrinsic resistances present among taxa that are part of stable microbial communities that help resist pathogenic invasion (Crits-Christoph et al., 2022; Moradi et al., 2022). Rather than signaling a harmful shift, the presence of these genes might reflect a microbiome that’s been shaped by past antibiotic exposure but remains functionally resilient (Bhattarai et al., 2024; Fishbein et al., 2023). This highlights an important point: the presence of resistance genes doesn’t always equate to increased infection risk; it depends heavily on the microbial community they’re part of and the ecological context in which they persist.

Our predictive model represents an advancement beyond existing approaches by simultaneously assessing the risk of both antibiotic-resistant infection and gastrointestinal colonization. This dual focus is relatively uncommon in current models, which often concentrate solely on infection outcomes, specific types of infections, or specific pathogens (Rich et al., 2022; Z. Wang et al., 2024). Our model’s design encompasses pathogens deemed as serious threats according to the CDC and various infection types, moving beyond the typical focus on single-species predictions. While our model yields a moderate AUC of 0.730, this performance is comparable to or exceeds that of other recent predictive models for ARI, where AUCs range from 0.57 to 0.75 (Liao et al., 2023; Rich et al., 2022). Additionally, the model achieved a Youden Index of 0.863, reflecting a strong overall balance between sensitivity and specificity. However, certain limitations warrant discussion. First, it is possible that utilizing a 16S rRNA sequencing-based abundance threshold at the genus level to determine which longitudinal stools to perform selective and differential culturing on might have led to missed ARC events. Moreover, it is also important to note that although combining colonization and infection events may obscure biologically distinct processes, this choice was made to not only better power analyses, but because it is commonly believed that bloodstream infections may stem from gastrointestinal colonization and translocation, making it biologically relevant to include these categories together. Second, while aggregating SHAP values across multiple independently trained models enhances the robustness of feature importance estimates, this approach can also amplify ambiguity, particularly when the underlying data relationships are complex. In this study, the variables utilized were microbial, which are known to exhibit extensive feature interactions and nonlinear relationships. Additionally, microbial taxa data are inherently zero-inflated, which contributes to some of the ambiguity in the SHAP interpretation, as reflected in the SHAP beeswarm plot where most data points are colored purple, indicating low or absent abundance for most taxa. These characteristics likely contribute to the observed ambiguity in SHAP results, as the effect of individual taxa on model predictions may vary depending on the presence and absence of other taxa and may not follow simple monotonic patterns. Nevertheless, the results in the SHAP plot are corroborated by those in the network plot, supporting the overall validity of our findings. Lastly, while our model demonstrates a notable improvement over existing tools, we recognize that our small cohort size, number of events, and single institution analyses are limiting the efficacy of our model. This model is in its developmental stages and further studies will be necessary to validate and refine this model in larger, multicenter cohorts and extend the analysis to different high-risk populations to enhance clinical applicability. Unfortunately, we have not been able to validate our model on an independent cohort, due to the inability to find a similar clinical cohort with the same sequencing and clinical metadata available.

In conclusion, this study provides an improvement in the predictive modeling of AR events in AML patients by integrating comprehensive microbiome, resistome, and clinical data. Although, in the early stages of model development, the performance of this model suggests a possibility for clinical utility and highlights its potential for future implementation in infection risk stratification once the limitations are addressed. These findings not only offer valuable predictors for clinical decision-making but also emphasize the critical importance of microbiome preservation during antibiotic therapy. Ongoing research and validation in diverse clinical populations will be essential to refine and implement these predictive tools effectively in clinical practice. Moreover, functional validation via metatranscriptomics, metabolomics, or gnotobiotic models would be valuable to determine the role of the predicted protective taxa, their metabolic products, and contributory ARGs on AR event risk.

## Data Availability

The datasets presented in this study can be found in online repositories. The names of the repository/repositories and accession number(s) can be found in the article/**Supplementary Material**.
