# Supplementary material for: Utilization of machine learning to predict antibiotic resistant event outcomes in acute myeloid leukemia patients undergoing induction chemotherapy
Source: Front Cell Infect Microbiol. 2025 Aug 21;15:1629422. doi: 10.3389/fcimb.2025.1629422 (PMC12408608; doi:10.3389/fcimb.2025.1629422)
Supplement: Supplementary file 1 [file DataSheet1.docx]

Supplementary Material

# Supplementary Data

# Supplementary Figures and Tables

## Supplementary Figures


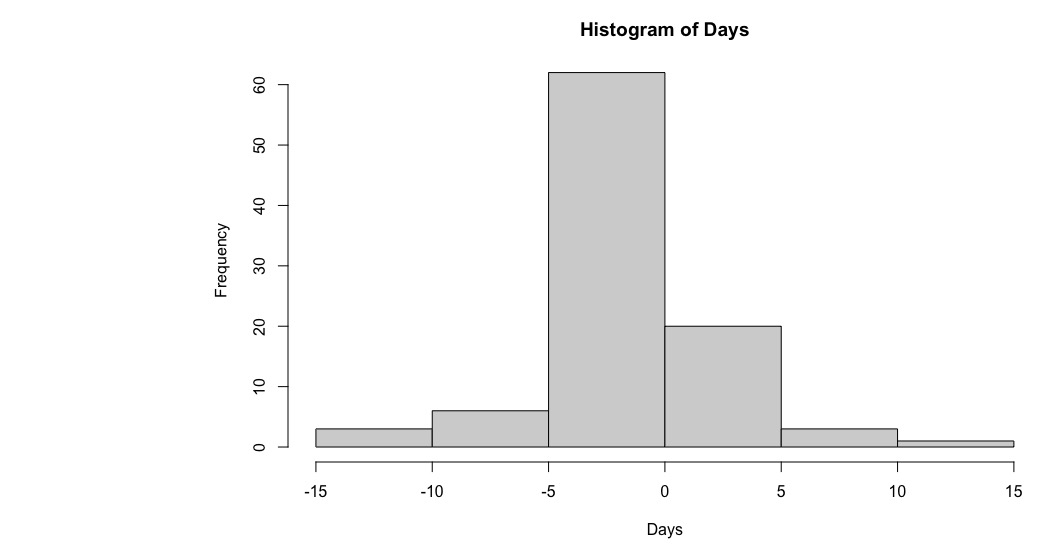


**Supplemental Figure 1. Distribution of Baseline Stool Sample Collection Dates**

The histogram depicts the number of days from initiation of chemotherapy that a patient’s baseline stool sample was collected. The average time to collection was 0.8 days before start of chemotherapy, and the range of collection was from 12 days prior to 11 days after the start of chemotherapy.

##
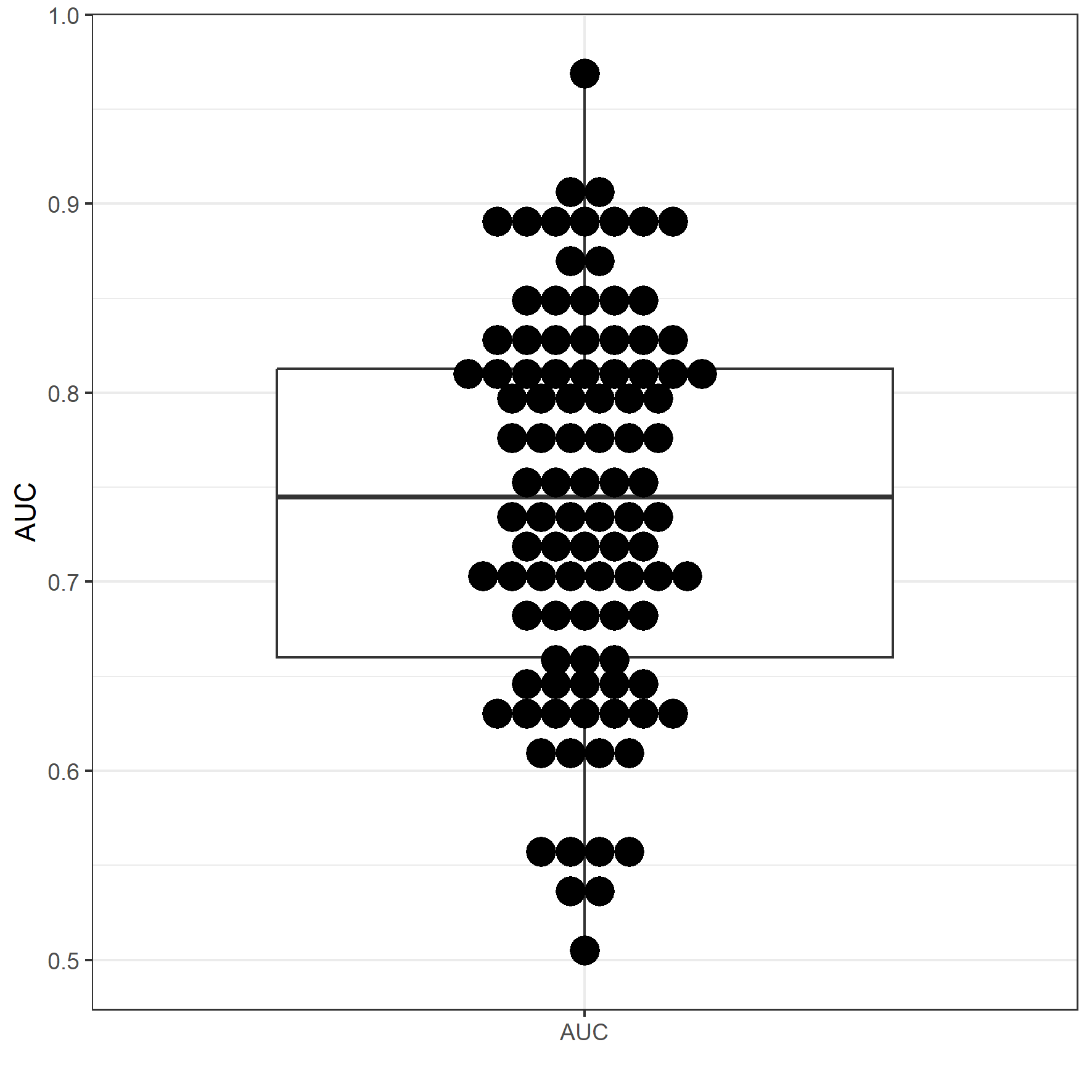


## Supplementary Figure 2. Distribution of Area Under the Curve (AUC) values from Random Forest Model 2 after optimization. (A) 100 iterations of the model were run, resulting in the distribution of AUC values shown in the box plot. The mean of the aggregates was 0.742, the median 0.745, and the AUC values ranged from a minimum of 0.505 to 0.969.

**
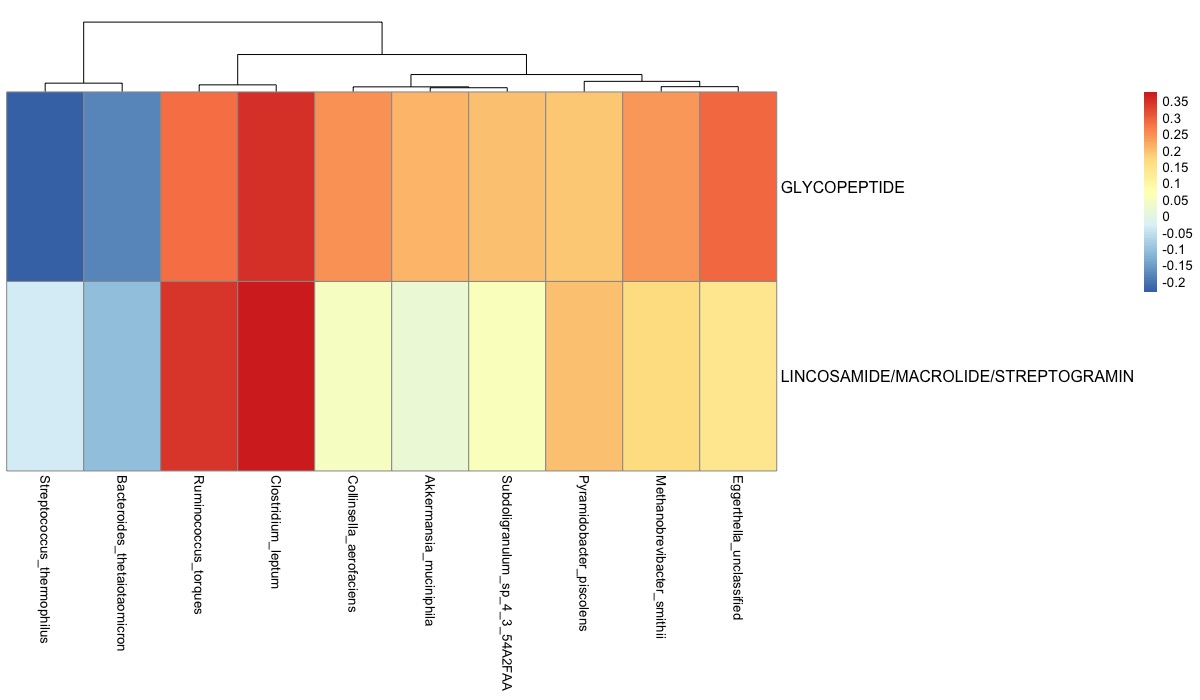
**

**Supplemental Figure 3. Sparse Canonical Correlation Analysis between Resistome and Microbiome Data.** (A) Heatmap correlogram showing Pearson correlation coefficient between gut microbiota species and classes of antibiotic resistance genes remaining after sparse canonical correlation analyses.

**Supplemental Table 1: Patients with AR-Event Outcomes and the Classification**.

| Cohort | Patient Number | Colonizing Species | Infectious Species | Colonization Classification | Infection Classification |
| --- | --- | --- | --- | --- | --- |
| PA13 | 2 | *E. cloacae, K. pneumoniae* | *P. aeruginosa, S. aureus* | CRE+, ESBL+ | CRE+, MRSA |
| PA13 | 7 | *K. pneumoniae, P. aeruginosa* | *P. aeruginosa* | ESBL+, CRE+ | CRE+ |
| PA13 | 41 | NA | *P. aeruginosa* | NA | CRE+ |
| PA13 | 45 | *E. faecium* | NA | VRE+ | NA |
| PA13 | 78 | *E. faecium* | NA | VRE+ | NA |
| PA13 | 81 | *E. cloacae* | NA | ESBL+ & CRE+ | NA |
| PA15 | 2 | *E. coli* | *E. coli* | ESBL+ | ESBL+ |
| PA15 | 5 | *S. aureus* | NA | MRSA+ | NA |
| PA15 | 13 | *E. coli, S. aureus* | NA | ESBL+, MRSA+ | NA |
| PA15 | 21 | NA | *E. coli* | NA | ESBL+ |
| PA15 | 26 | NA | *E. coli* | NA | ESBL+ |
| PA15 | 29 | NA | *E. coli* | NA | ESBL+ |
| PA15 | 35 | *S. aureus* | NA | MRSA+ | NA |
| PA15 | 46 | NA | *E. coli* | NA | ESBL+ |

**Supplemental Table 2: Clinical Metrics for AR Models**

|  | **Model 1** | **Model 2** | **Model 3** | **Model 4** | **Model 2 Optimized Aggregate** |
| --- | --- | --- | --- | --- | --- |
| **Sensitivity** | 0.785 | 0.630 | 0.778 | 0.693 | 0.685 |
| **Specificity** | 0.503 | 0.643 | 0.522 | 0.588 | 0.6662 |
| **PPV** | 0.208 | 0.227 | 0.213 | 0.219 | 0.253 |
| **NPV** | 0.933 | 0.913 | 0.934 | 0.920 | 0.927 |
| **Youden Index** | 0.879 | 0.835 | 0.871 | 0.855 | 0.863 |

**Supplemental Table 3: Confusion Matrices for AR Models at Optimal Youden Threshold**

|  |  | Predicted Negative | Predicted Positive |
| --- | --- | --- | --- |
| Model 1 | Actual Negative | 1194 | 314 |
|  | Actual Positive | 1206 | 86 |
|  |  |  |  |
|  |  | Predicted Negative | Predicted Positive |
| Model 2 | Actual Negative | 857 | 252 |
|  | Actual Positive | 1543 | 148 |
|  |  |  |  |
|  |  | Predicted Negative | Predicted Positive |
| Model 3 | Actual Negative | 1147 | 311 |
|  | Actual Positive | 1253 | 89 |
|  |  |  |  |
|  |  | Predicted Negative | Predicted Positive |
| Model 4 | Actual Negative | 988 | 277 |
|  | Actual Positive | 1253 | 89 |
|  |  |  |  |
|  |  | Predicted Negative | Predicted Positive |
| Model 2 *Optimized Aggregate Model* | Actual Negative | 811 | 274 |
|  | Actual Positive | 1589 | 126 |

**Supplemental Table 4: Network Primary Node Connections to AR-Event Outcome**

| Node | Weight | Correlation to AR Event |
| --- | --- | --- |
| Methanobrevibacter smithii | 0.3120369 | Negative |
| Bacteroides massiliensis | 0.3094032 | Negative |
| LINCOSAMIDE/MACROLIDE/STREPTOGRAMIN | 0.25323823 | Negative |
| Bacteroides dorei | 0.22755376 | Negative |
| Dorea longicatena | 0.22265911 | Negative |
| Clostridium leptum | 0.22070903 | Negative |
| Streptococcus mutans | 0.20965496 | Negative |
| Eubacterium siraeum | 0.20619186 | Negative |
| Dialister invisus | 0.20616364 | Negative |
| Barnesiella intestinihominis | 0.20364759 | Negative |
